# Feeling happy and carefree: a qualitative study on the experiences of parents, medical clowns and healthcare professionals with medical clowns

**DOI:** 10.1080/17482631.2018.1503909

**Published:** 2018-08-29

**Authors:** Jesminne Bruins Slot, Michelle Hendriks, Ronald Batenburg

**Affiliations:** a Care from the Patient Perspective, Netherlands Institute for Health Services Research (NIVEL), Utrecht, the Netherlands; b Professions in Health Care and Manpower Planning, Netherlands Institute for Health Services Research (NIVEL), Utrecht, the Netherlands

**Keywords:** Clown doctors, medical clowns, disabled children, hospitalized children, qualitative methods

## Abstract

**Purpose:** To explore the effect of medical clowns and its relevant actors and conditions.

**Method:** Semi-structured interviews were conducted with fourteen parents who had experiences with medical clowns in the Netherlands. Four focus groups were held with seven medical clowns and 25 healthcare professionals. The interviews and focus groups were audio-recorded and transcribed verbatim. Content analysis was used to analyse the data.

**Results:** Concerning the clown effect, we distinguished the following themes: happiness, distraction, carefree feeling and activation. This effect depended upon clown characteristics (appearance, tailoring, low-key play, making an effort); child characteristics (age, autonomy, fear, living cut off from society, communication); parent characteristics (autonomy, attitude); healthcare professional characteristics (attitude, communication); and organizational conditions (timing and planning, collaboration, accessibility, awareness).

**Conclusion:** This study shows that medical clowns are of value for children with serious illnesses or mental disabilities in several care settings. An asset of medical clowns is that they tailor their play to the child and situation.Support of and communication with parents and healthcare professionals is critical. The proposed model of the clown effect can help shape future research. The results can help medical clown organizations to enhance their services and optimize clown encounters.

## Introduction

Children with a chronic illness or mental disability often face physical, emotional and psychosocial challenges (Rindstedt, 2014). They spend long days in hospitals, go to special centres for children with disabilities and/or are separated from their normal environment with relatives and friends. Medical clowns can support these children, promote recovery and minimize stress (Pendzik & Raviv, 2011). They use a variety of activities such as music, magic, play, and joke telling (Armfield, Bradford, White, Spitzer, & Smith, 2011).

**Figure 1.**
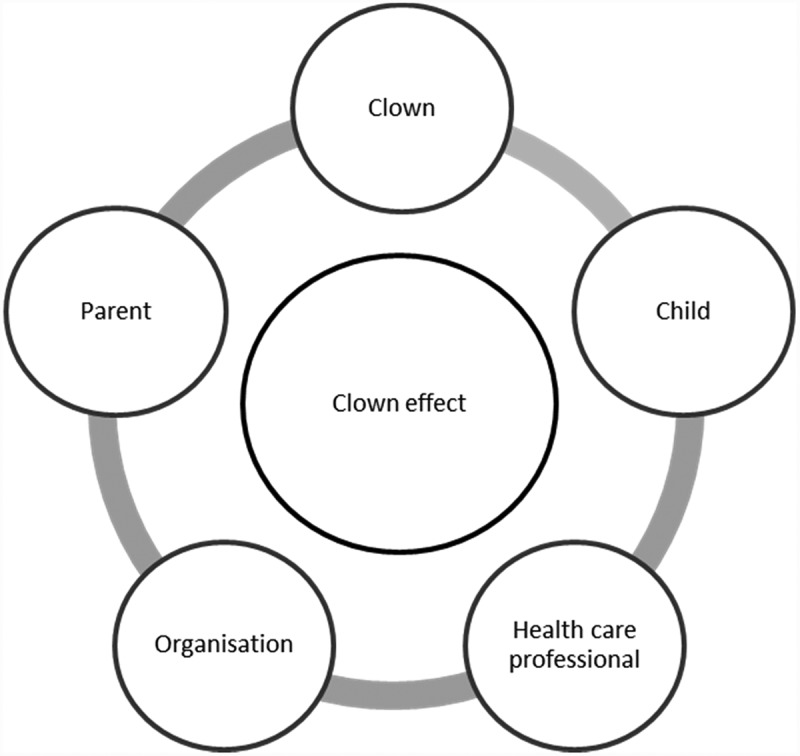
Model of actors and conditions related to the effect of medical clowns.

The presence of a clown has been found to reduce the child’s anxiety (Dionigi, Sangiorgi, & Flangini, 2014; Felluga et al., 2016; Goldberg et al., 2014; Kocherov et al., 2016; Meiri, Ankri, Hamad-Saied, Konopnicki, & Pillar, 2016; Messina et al., 2014; Vagnoli, Caprilli, & Messeri, 2010; Vagnoli, Caprilli, Robiglio, & Messeri, 2005; Weintraub et al., 2014) and feelings of boredom, loneliness and sadness (Finlay, Baverstock, & Lenton, 2014; Mansson, Elfving, Petersson, Wahl, & Tunell, 2013; Meiri et al., 2016). Also, a few studies showed that a clown intervention is able to reduce anxiety in parents (Agostini et al., 2014; Fernandes & Arriaga, 2010; Wolyniez et al., 2013). Other studies show more mixed results. In the study by Golan et al., clown intervention did reduce anxiety levels while children were waiting for anaesthesia and surgery (Golan, Tighe, Dobija, Perel, & Keidan, 2009), but when the aesthetic mask was applied, anxiety levels increased to a higher level than in children in the control group. Hansen et al. found a significant positive effect of clown presence on girls, but not on boys (Hansen, Kibaek, Martinussen, Kragh, & Hejl, 2011). Studies so far mainly looked at the effect of clown visits on children who had to undergo surgery or invasive procedures and their parents. In recent reviews, it was concluded that medical clowns help in reducing psychological distress, anxiety and pain in these children and their parents (Phal, Tardieu, ALessandrini, Gentile, & Rindstedt, 2018; Sridharan & Sivaramakrishnan, 2016; Zhang, Yang, Lay, Garg, & Lao, 2017).

Several qualitative studies have investigated the determinants of the clown effect. Children, family and healthcare professionals described the clown visits as moments of fun and distraction (Ford, Courtney-Pratt, Tesch, & Johnson, 2014; Ofir, Tener, Lev-Wiesel, On, & Lang-Franco, 2016). The clowns tailored their play to the situation, created a bond with the child and reframed the meaning of the medical procedures. Mansson et al. (2013) found that clowns helped to reduce boredom and homesickness, but that children did not want a clown present during tests or medical procedures.

To date, studies mainly focused on clown activities for children in hospitals. Whether medical clowns can be of value in other care settings and for other groups of children is largely unknown. To our knowledge, there is only one study that explored the response of children with mental disabilities to therapeutic clowns (Kingsnorth, Blain, & McKeever, 2011). Furthermore, there are only a few studies on how clowns themselves value their work (Linge, 2008) and the experiences of healthcare professionals (Barkmann, Siem, Wessolowski, & Schulte-Markwort, 2013; Blain, Kingsnorth, Stephens, & McKeever, 2012; Ford et al., 2014; Linge, 2011). For example, clowning may improve communication among healthcare professionals and affect the nurse–patient relationship (Blain et al., 2012). Healthcare professionals are also very important in determining whether the clown visit will succeed. For example, in the study of Vagnoli et al. (2005), hospital staff opposed the presence of clowns before surgery.

In the Netherlands, medical clowns known as “CliniClowns” visit children not only in hospitals but also in rehabilitation centres and centres and schools for mentally disabled children. They organize special events such as circuses and provide online activities. The CliniClowns Foundation states that it is important that the clowns’ activities meet the needs of all parties (children, parents, healthcare professionals and clowns). Therefore, we explored the experiences of parents, medical clowns and healthcare professionals with the activities of medical clowns in the Netherlands. This study adds to the literature by examining the clown effect and its relevant actors and conditions, by looking at experiences in care settings other than hospitals and by involving different parties.

## Methods

### Study design

Based on the literature, we created a model to depict the clown effect with its relevant actors and conditions (see Figure 1). The clown effect is the effect that a clown can have on children, parents and healthcare professionals. The following actors or conditions influence and/or are influenced by the clown effect: child as main recipient, clown, parent or caregiver, healthcare professionals and organization of clown activities.

We performed a qualitative study between October 2015 and February 2016. Few studies so far have focused on the determinants of the clown effect. Also, the views of parents, medical staff and medical clowns have hardly been examined. We therefore chose to perform a qualitative study to get insight into all relevant actors and conditions. We choose to interview parents individually to provide them with plenty room to tell their own story. This also made it possible to interview parents at home, increasing the response rate. We interviewed the parents rather than the children because we were also interested in the clown effect on children too young or too disabled to communicate their experiences. Children were free to be present during the interview and tell their story. Clowns and healthcare professionals were interviewed in focus groups making it possible to discuss the factors that enable and hinder a successful clown visit.

### Participants

Parents and other caregivers were recruited via a post on the web site and Facebook page of the CliniClowns Foundation. Among those who volunteered, we selected parents/caregivers who had had experience with CliniClowns within the last year and as a group covered a broad spectrum of CliniClowns activities. Halfway through the interviews, we observed that most participants only reported positive experiences. In order to provide a full picture and increase the diversity in participants, we recruited one parent who was known to be less positive about the CliniClowns by purposive sampling.

We organized four focus groups with four distinct groups: medical clowns, pedagogical staff in hospitals, healthcare professionals for long-term and chronic care (doctors, nurses and teachers), and healthcare professionals working with mentally disabled children (day-care centre employees). The CliniClowns Foundation recruited healthcare professionals and clowns from different regions and settings using their network (purposive sampling). We also placed a post on the NIVEL web site inviting healthcare professionals and spread this invitation by NIVEL social media, but nobody responded. NIVEL is the Netherlands institute for health services research.

### Interview and focus groups

Semi-structured interviews were conducted with parents about their experiences with the different clown activities. The focus groups were semi-structured and groupware software was used to elicit the participants’ opinions on different topics. The discussion was divided into different rounds. At the beginning of each round, participants answered a few questions on a laptop. The results were projected onto a screen and discussed with the group. An advantage of this procedure is that it enables a balance between group dynamics and the collection of individual experiences (Batenburg & Bongers, 2001). See Table 1 for the topic lists for the interviews and focus groups.

**Table 1.** Topic lists for the interviews with parents and focus groups with clowns and healthcare professionals.

**Topic list for interviews with parents**
1. Child’s problem
2. CliniClown activities that parents have experience with
3. Description of the encounters with the CliniClowns:
– Clown characteristics
– Child’s response
– Response of the parent/caregiver
– If applicable: differences between different encounters/activities
– Effect on parent–child relationship
4. Positive aspects of clown activities
5. Negative aspects of clown activities (room for improvement)
6. Added value of CliniClowns compared with alternatives (for instance, pedagogical staff)
7. When can children benefit from a clown performance? What groups of children?
**Topic list for focus groups with clowns and healthcare professionals**
1. Round 1: introduction
– Years working as/with CliniClowns
– Experiences with/familiarity with different clown activities
2. Round 2: experiences with CliniClowns
– Example of successful clown performance, that is an example in which the clowns had a positive effect
– Example of unsuccessful clown performance
3. Round 3: facilitators and barriers
– What is important for a successful clown performance?
– What is important for cooperation between clowns and healthcare professionals?
4. Round 4: target groups
– What groups of children can benefit from a clown performance
5. Round 5: the future
– Where or when do CliniClowns have to stay?
– Where or when are CliniClowns missed?

JB conducted the semi-structured interviews with parents. All authors took part in the focus groups. All authors had no experience with CliniClowns themselves. Besides having knowledge of the existing literature, they had no biases regarding the clown effect and relevant actors and conditions. The parent that was included by purposive sampling was a colleague of the authors, which is why we knew she was not positive about the clown visits.

### Analysis of data

The interviews and focus groups were recorded with permission of the participants, and transcribed verbatim. No names were mentioned in the transcripts. If participants used names during the interviews, these were abbreviated to the first letter in the transcripts. Both the audio records and transcripts were only accessible for the researchers in the project team. After the study, the transcripts were shared with CliniClowns Foundation with written consent of the parents/caregivers and verbal consent of the other participants.

The transcripts were coded with MaxQDA. The constant comparative method (CCM), a core analysis technique in the grounded theory approach (Boeije, 2002), was used to analyse the data. After three single interviews were read and open-coded by two researchers independently (JB and MH), the coded transcripts were compared, and a first code tree was created. We organized the themes according to the proposed model of the clown effect as much as possible. Next, all interviews and focus groups were coded using this code tree as basis. Two researchers (JB and MH) compared a selection of three interviews that they had coded separately and discussed whether the code tree needed adjustments. Several small changes were made. Almost all coded parts were the same. Where codes were different, the two researchers reached consensus without having to consult a third person.

## Results

### Respondents

Fourteen participants were interviewed in 12 interviews by JB. One interview was with a grandparent, who was also the child’s caregiver; the other participants were all parents. For readability, we will refer to this group as “parents”. Most parents had experiences with clown visits in the hospital, had visited a CliniClowns activity or had had the CliniClowns visit their child’s school or day-care centre. Only three parents talked about their experiences with online activities (see Table 2). The interviews lasted between 30 min and 1 h.

**Table 2.** Number of participants having experience with the different types of clown activities.

Activities	(Grand)parents *n* = 14	Clowns *n* = 7	Pedagogic staff in hospitals *n* = 7	Healthcare professionals for mentally disabled children (day-care centre employees) *n* = 10	Healthcare professionals for long-term and chronic care (doctors, nurses and teachers) *n* = 8
CliniClowns in hospital	10	6	7	1	7
*Clowns visit children’s wards in hospital. Main service: two clowns perform for each child individually*					
Attending a CliniClowns activity	8	6	2	5	4
*Children and their parents attend an activity, for example the CliniClowns circus or college*					
Performance in a school or day-care centre for children with disabilities	7	7	1	9	4
*CliniClowns perform for a group of children*					
Online CliniClowns Club	3	2	0	0	0
*CliniClowns have an online community where children can watch movies and interact with CliniClowns and other children*					

The number of participants in the focus groups ranged from seven to 10 (see Table 2). The participants covered the whole spectrum of CliniClowns activities. However, only three clowns had participated in online activities, and the healthcare professionals were hardly familiar with the online activities. All focus groups lasted about 2 h.

Below, we describe the results of both the interviews and focus groups together using the proposed model of the clown effect.

### Clown effect

We extracted four themes that describe the clown effect: *happiness, distraction, carefree feeling* and *activation*. Most participants said that the medical clowns made the children happy and joyful.

All her facial expressions show it (…) She is now in a customized buggy and you can put a brake on it, but that is of no use (…). She is just unable to sit still because of her enthusiasm. (parent 5)

Parents had the feeling that the clowns were there not only for their child, but also to support the parents and make them feel better.

On the ICU, when a child is sedated or kept asleep, he [the child] doesn’t know who’s at his bedside. And then they [the clowns] still visit. And as a parent you know darn well that they are there for you. (…) It just cleared the air, it has nothing to do with being sick, it’s a laugh and a bit of distraction. (parent 3)

Also, the clowns could *distract* the child and parents during the long days in the hospital and the unpleasant and boring periods.

He was in a small room and he couldn’t leave the room. Then you spend four weeks in a space of only eight square metres. (…) A visit by a CliniClown makes you forget this situation for a while. (parent 3)

Parents with a child with a mental disability pointed out that they experienced a *carefree feeling* when they attended activities of the CliniClowns. They felt they were among peers and that did not have to inhibit their child’s behaviour.

When we go to a regular circus, (…) then you often feel a bit embarrassed, because you bother other people (…) And here [CliniClowns activity] you don’t. I mean, all the children are doing something inappropriate, so she can really be herself and we can pay less attention to what she does and how she does it. (parent 5)

Some parents, some clowns and many healthcare professionals mentioned that the clowns could provoke behaviour that nobody expected from the child – that the clown was able to *activate* the child. While the people around the child mainly focused on what the child could not do or should try to do, the clown showed what the child was capable of doing, amazing parents and healthcare professionals.

Doctors enter with “we have a problem”. The nursing staff come in with “we must solve the problem” and we come in with “Hi, what’s possible?!” (…) And then (…) the parents or the child themselves discover what they still can do thanks to our visit. (clown 7)

### Clown characteristics

According to the participants, medical clowns had particular characteristics and skills that distinguished them from other clowns, entertainers or healthcare professionals. We found four themes: *appearance, tailoring, low-key play* and *making an effort.*

Parents noted that the clowns wore a red nose and colourful clothes, but their faces were not painted. This made the clowns funny-looking and less scary for the children. It was important that the clowns were recognizable as such, because people have different expectations of a clown than of an ordinary person or healthcare professionals.

The clown can behave a bit crazy, but if someone, (…) wears a tie and we don’t know that person (…) and he behaves a little crazy, then I’m like “friend, it’s time for you to go”. (parent 6)

The different expectations are strengthened by the fact that the clown does not visit the hospital every day and is not part of the medical staff. Both aspects were valued by the parents.

Pedagogical staff can also say “I’ll put your drip down over here, (…) and the nurse will come soon and do this and that …” CliniClowns will never talk about that. They won’t say if the pump beeps, “Oh the pump’s beeping, we have to call a nurse”. Perhaps they’ll imitate the sound of the pump, but they won’t be a part of the medical scene. (parent 3)

All groups said that the clowns *tailored their play* to the child and situation. Medical clowns studied and mimicked the child, but also had an eye for the environment.

They really watch the child. Again it’s that attention and the patience and looking what the child can do, and what they respond to. (parent 11)

One of the most frequently mentioned characteristic was that the clowns used *low-key play*. The play was not exaggerated and overwhelming; it focused mostly on stimulating the different senses.

These two clowns are more low-key in how they play, it’s not loud, they don’t shout in the hallway or run around with toilet paper or something. They play on a small scale, with songs and a cuddly toy. (pedagogic worker 7)

The combination of tailoring and low-key play makes parents and healthcare professionals label clowns as professionals. Medical clowns are different from ordinary clowns and other performers in this way.

Some interviewees also mentioned that the clowns *made an extra effort* when needed. For example, when children were in isolation, some clowns were inventive and made a whole play behind the window of the isolation room or donned the special clothes so they could enter the room.

She was so unlucky she even caught swine flu. And even then, they [the clowns] came. They just put on a mouth cap and put on gloves and started to play. (…) That’s hats off to them. (parent 6)

### Child characteristics

For the actor child, we distilled the themes *age, autonomy, fear, living cut off from society* and *communication*. There were discussions about the maximum *age* at which medical clowns could still be of value. While some parents said that clowns were less interesting for teenagers, others said clowns could be for all ages. Children with a mental disability in particular would probably never outgrow the clowns. Parents and healthcare professionals indicated that it is more important to look at someone’s developmental level than their age. Regarding children with a mental disability:

Only the body gets bigger, the experience with the CliniClowns stays the same. (pedagogic worker for children with mental disabilities 2)

Another theme that emerged in the interviews was respect for the child’s *autonomy*. Most parents said that the autonomy of their child was respected or even enhanced. The child was in charge, he or she could decide whether the clown should visit and what the visit should be like.

A child went for an operation and was scared. Then they [CliniClowns] walked all the way to the operating room, in a kind of procession. But the child was in charge, something that’s rare at such moments. (pedagogic worker hospital 3)

However, one parent told that her child’s autonomy was not respected. This child did not like the clowns, but the healthcare professional asked the child to cooperate with the clowns anyway. The clowns mentioned that sometimes healthcare professionals urged them to visit children who were unwilling and this was detrimental for the visit. Also, as pointed out by both healthcare professionals and clowns, some children have a *fear* for clowns, and in these cases it is important to respect the wishes of the child.

When asked which children can benefit from medical clowns and which groups are potential new clients for CliniClowns, clowns and healthcare professionals suggested that children *living cut off from society* and children who have difficulties *communicating* verbally would benefit in particular.

Children who go to school (…) take part in society, whereas if you are in hospital or you can’t go out because you’re sick (…) then you are so cut off from the rest of the world. (teacher of children with mental disabilities 3)

Groups that were mentioned were refugee children, children receiving home care, children with psychiatric or behavioural problems and children in shelters for battered woman. Children in asylum centres were thought to benefit:

Because you don’t have to speak the language to make contact with them. (hospital nurse 2)

Nevertheless, the consensus was that medical clowns were needed most in hospitals.

### Parent characteristics

Two themes were found for the actor parent, *autonomy* and *attitude*. The autonomy of the parents should also be respected. One mother said that the medical clowns did not listen to her wishes.

I had closed the door. The curtains were also closed. (…) they [the clowns] still knocked on the door. We didn’t respond, but they knocked again. So I opened the door, and they [the clowns] said. “No, they don’t want a clown”. I closed the door. They opened the door again: “They really don’t want it, they don’t want it at all, do they?” (…) And that wasn’t just one incident, it happened more often. (parent 12)

In the focus group with clowns, it became evident that clowns are not always appreciated by the parents. The *attitude* of the parents is therefore an important aspect in determining whether the clowns can reach the child.

The father wanted us out and didn’t give any space for his son. At a certain point this can become awful, because you don’t know if the son wants you there or not. (clown 2)

### Healthcare professional characteristics

Two themes were found for healthcare professionals: *attitude* and *communication*. Clowns and healthcare professionals mentioned that the *attitude* of the healthcare professionals is a key factor determining whether the clowns are welcome or not. *Communication* between the clowns and staff about the clowns’ methods and how staff can deal with clowns might help to overcome a negative attitude among healthcare professionals.

The staff had difficulties with how to respond. (…) So the CliniClowns gave a training course (…). [They explained about] the contact between them and the children, and that the nursing staff sometimes walked straight through it, and that that made them lose contact with the child. (…) That worked well. (child hospice nurse)

### Organizational factors

We found four themes regarding the organization: *timing and planning, collaboration, accessibility* and *awareness of activities*. The clowns visit each hospital once or twice a week and centres or schools for disabled children once or twice a year. These centres or schools also get the opportunity to go to the circus once or twice a year. Most participants liked this frequency, but some thought it could be more. Some parents planned hospital visits on days they knew the medical clowns would be there. A disadvantage of a set day or time is that the child will miss the clowns if he/she is not at the location when the clowns visit.

Unfortunately, M. [the child] hasn’t experienced a clown visit yet [in the day-care centre], because they always visit on days she’s not there. I think that’s a real pity, I think they visit only once a year. (…) They shouldn’t visit every week, then it wouldn’t be special anymore, but they should come at least once a quarter or so. (parent 11)

*Collaboration* between healthcare professionals and the clowns was an important theme. The clowns’ visits were organized differently in different hospitals. In some hospitals, the pedagogical staff and the clowns discussed beforehand which children would be visited that day, while in other hospitals, clowns were not briefed at all. Both medical clowns and healthcare professionals mentioned that it is important that clown and hospital professionals know what they can expect from each other:

If I were a CliniClown I could imagine that it could be a barrier if you walk into a new ward and you don’t know what the agreements are. [It’s important] (…) for the nursing staff to know what they [the clowns] will do. (hospital nurse 1).

In the Netherlands, the CliniClowns’ activities are free of charge. This makes the activities financially *accessible* to everyone. Some parents said that the CliniClowns circus and college were very popular, which make it difficult to get tickets. However, the clowns did not recognize this. It became evident that the parents and healthcare professionals were *not aware of all the available activities*. Everyone knew that the clowns play in hospitals, but parents and healthcare professionals were less aware of activities such as the circus and online activities.

## Discussion

This study confirmed that medical clowns can have a positive effect on children, parents and healthcare professionals in a hospital environment. We expanded on previous knowledge by showing that medical clowns are also of value in other care settings, such as rehabilitation centres, day-care centres and schools for children with mental disabilities. Parents, medical clowns and healthcare professionals reported positive experiences with visits by medical clowns, but also with other activities such as circuses and online activities. Several actors and conditions were distinguished that determined these positive experiences and the clown effect.

Concerning the clown effect, parents, medical clowns and healthcare professionals mentioned that clowns bring joy and distraction to both children and parents, that they are able to activate children and make them forget their illness or disability for a while providing a carefree moment. While the care settings and activities differ, the following clown characteristics were mentioned as strengths in all groups of participants: medical clowns employ a low-key style of play, make an extra effort for the child and are able to tailor their performance to the child and the situation. Linge described this as the “optimal encounter”. In an optimal encounter, the child is acknowledged and has the chance to express themselves in interactive play with the clowns (Linge, 2008).

Following the proposed clown model, several characteristics of the child, parents and healthcare professionals were distinguished that influenced the clown effect. Participants indicated that it is important for all parties to have a positive attitude towards the clown encounter and for the autonomy of both child and parent to be respected. In our study, one parent told how her child did not enjoy the clown visits. Other studies also showed that not all children like medical clowns (Ford et al., 2014) and that some children are even afraid of them (Meiri et al., 2017). This should be taken into account when medical clowns decide which children to visit.

When participants were asked what groups of children could benefit from the medical clowns, they said that children living cut off from society and children who have difficulties communicating verbally could benefit from a visit by medical clowns. People aged over 18 with mental disabilities, children recovering from an illness at home and refugee children were mentioned most often as other groups that could benefit from clown visits. It would be interesting to investigate if medical clowns are of value for these groups. There is one study that investigated the effects of teleclowning on children being cared for at home (Armfield et al., 2011). Clowning at a distance appeared to be technically and practically feasible, and the clowns were able to develop a relationship with the children. In the future, teleclowning might be a suitable clown intervention for children receiving care at home.

As found by Blain et al. (2012) and Van Venrooij and Barnhoorn (2017), the clown effect is enhanced by close collaboration and communication between medical clowns and healthcare professionals. In other countries, for instance Israel, medical clowns are involved in medical procedures, and they cooperate closely with hospital staff. Studies have shown positive effects of these activities on the children, and it has been suggested that medical clowns should become an integral part of the health team (Kocherov et al., 2016; Ofir et al., 2016; Tener, Ofir, Lev-Wiesel, Franco, & On, 2016; Wolyniez et al., 2013). However, other studies showed a more negative attitude and suggested that the presence of clowns may interfere with medical routines and that children did not want a clown present during tests or medical procedures (Mansson et al., 2013; Vagnoli et al., 2005; Van Venrooij & Barnhoorn, 2017). In our study, most participants also did not support the idea of medical clowns being present during medical procedures. They felt it was a good thing that the medical clowns are not part of the hospital organization. The differences in results might be explained by the fact that Dutch hospitals have pedagogical staff to support and distract the child during medical procedures, which is not the case in most other countries. This can make it difficult to translate findings from studies abroad to the Dutch situation and vice versa.

Concerning the organization of the clown visits, one interesting finding was that most parents and healthcare professionals did not know about the full range of CliniClowns activities. People are familiar with the visits to hospitals, but activities such as the circus and online activities were less well known. As a consequence, children may miss out on encounters with the medical clowns. More publicity for the different activities could overcome this. In addition, it was said that the frequency of visits should be high enough to give all children the opportunity to meet the medical clowns but low enough to keep the encounters special. The optimal frequency is yet unknown, and it probably differs between healthcare settings. In a study among parents of children admitted to the intensive care, nine parents (27.3%) suggested clowns play at the bedside daily, 17 (51.5%) suggested twice a week, and seven (27.3%) suggested once a week (Mortamet et al., 2017). This issue should be addressed in future research.

This study has some limitations. Participants were recruited via the CliniClowns Foundation, which may have led to a bias in favour of the clowns. The parents in particular reported only positive experiences with the medical clowns. Therefore, we recruited one parent we knew to be critical. In the focus groups with clowns and healthcare professionals, both more positive and less positive aspects of the clown encounters were mentioned. As there was a high degree of agreement across the different groups of participants and with previous studies, we feel confident that the results reflect the most relevant themes. Another limitation is that children were not interviewed in this study. We chose to interview the parents, as we were interested in experiences with the broad range of CliniClowns activities, including activities for children with a mental disability or very young children. It is hard for these children to express themselves in an interview.

## Conclusion

Medical clowns are of value in hospitals and other care settings, and for children with serious illnesses or with mental disabilities. Clown visits have several positive effects. Besides bringing joy, medical clowns are able to activate children and provide a carefree moment. Several characteristics of the clown, child, parents, healthcare professionals and organization appeared to determine these clown effects. An important factor is the ability of medical clowns to optimally tailor their performance to the child and situation. The proposed model of the clown effect can help shape future research. The results provide the Dutch Foundation for CliniClowns and medical clown organizations in other countries with suggestions on how to enhance their services and optimize clown encounters.
